# Performance of retinal fluid monitoring in OCT imaging by automated deep learning versus human expert grading in neovascular AMD

**DOI:** 10.1038/s41433-023-02615-8

**Published:** 2023-06-13

**Authors:** Maximilian Pawloff, Bianca S. Gerendas, Gabor Deak, Hrvoje Bogunovic, Anastasiia Gruber, Ursula Schmidt-Erfurth

**Affiliations:** Department of Ophthalmology, Medical University of Vienna, Vienna, Austria

**Keywords:** Retinal diseases, Medical imaging

## Abstract

**Purpose:**

To evaluate the reliability of automated fluid detection in identifying retinal fluid activity in OCT scans of patients treated with anti-VEGF therapy for neovascular age-related macular degeneration by correlating human expert and automated measurements with central retinal subfield thickness (CSFT) and fluid volume values.

**Methods:**

We utilized an automated deep learning approach to quantify macular fluid in SD-OCT volumes (Cirrus, Spectralis, Topcon) from patients of HAWK and HARRIER Studies. Three-dimensional volumes for IRF and SRF were measured at baseline and under therapy in the central millimeter and compared to fluid gradings, CSFT and foveal centerpoint thickness (CPT) values measured by the Vienna Reading Center.

**Results:**

41.906 SD-OCT volume scans were included into the analysis. Concordance between human expert grading and automated algorithm performance reached AUC values of 0.93/0.85 for IRF and 0.87 for SRF in HARRIER/HAWK in the central millimeter. IRF volumes showed a moderate correlation with CSFT at baseline (HAWK: r = 0.54; HARRIER: r = 0.62) and weaker correlation under therapy (HAWK: r = 0.44; HARRIER: r = 0.34). SRF and CSFT correlations were low at baseline (HAWK: r = 0.29; HARRIER: r = 0.22) and under therapy (HAWK: r = 0.38; HARRIER: r = 0.45). The residual standard error (IRF: 75.90 µm; SRF: 95.26 µm) and marginal residual standard deviations (IRF: 46.35 µm; SRF: 44.19 µm) of fluid volume were high compared to the range of CSFT values.

**Conclusion:**

Deep learning-based segmentation of retinal fluid performs reliably on OCT images. CSFT values are weak indicators for fluid activity in nAMD. Automated quantification of fluid types, highlight the potential of deep learning-based approaches to objectively monitor anti-VEGF therapy.

## Introduction

Age-related macular degeneration (AMD) accounts for 7% of global blindness and is one of the leading causes of irreversible vision loss in developed countries [1, 2]. On a global level, AMD affected over 170 million people [3] in 2018. Because of the rising prevalence of AMD as the worldwide population ages, 288 million people are projected to suffer from early or late stages of AMD by 2040 [4]. Late stages are characterized by significant loss of central vision due to either geographic atrophy or development of neovascularization.

Neovascular AMD is characterized by abnormal angiogenesis originating either from the choroid or, less frequently, from the retinal circulation [5]. These aberrant vessels are susceptible to leakage resulting in fluid accumulation within and underneath the retina, haemorrhaging and eventually fibrosis which leads to rapid visual loss compared to eyes with geographic atrophy, which have a more gradual decline of visual function. Although only about 15% of patients with AMD develop neovascularization, over 80% of cases of blindness were caused by the exudative type before the advent of vascular growth factor inhibitors [6] (VEGF). Anti-VEGF substances have introduced a new standard of care in the treatment of patients with nAMD [7], allowing for a substantial recovery with long-term stabilization of visual acuity [8] where previously extensive structural damage would inevitably occur. However, the enormous costs and labour of sustained therapy over lifetime of these vision-threatening diseases in the developed world place an exceptional strain on patients and healthcare systems [9]. Consecutively, real-world outcomes differ greatly from benefit levels achieved in clinical trials [10]. Although the amount of detail generated by high-resolution three-dimensional OCT imaging is enormous, the morphological information cannot be exploited sufficiently, as the large volumes make the manual analysis of pathological features of CNV time-consuming and impossible in real-world settings. For these reasons conventionally mostly qualitative aspects are assessed (e.g., presence or absence of IRF/SRF) and the few quantitative markers that do exist i.e. CSFT neither correlate with baseline visual acuity (VA) or VA after treatment (in the range of 0.2 to 0.3) or retinal fluid [11, 12]. In spite of this challenge, current clinical trials uniformly use treatment regimen based on central subfield thickness (CSFT) [13]. Therefore, an innovative approach to precisely and quantitatively measure clinically relevant features such as fluid in an automated fast and objective manner is needed to support clinicians in making reliable treatment decisions.

Deep learning-based algorithms have been proposed for the classification of intraretinal (IRF) and subretinal fluid (SRF) on a pixel by pixel basis and their accuracy will further improve in the near future. The focus of this study was to provide evidence that the performance of such an automated deep learning algorithm can be considered consistent with or even superior to the ground truth represented by measurements made by a certified reading centre in the relevant aspects of anti-VEGF monitoring: identifying retinal fluid presence or absence, evaluating fluid resolution under therapy fluid and correlating measured fluid volumes with the traditionally used marker CSFT in a large number of OCT scans of patients treated with an approved therapy for neovascular AMD (nAMD). Furthermore, we evaluated and compared thickness measurements from the automated layer boundary identification algorithm with centre point thickness (CPT) and CSFT measurements by the Vienna Reading Center representing the current gold standard measurements.

## Methods

This retrospective study was conducted in accordance with the Declaration of Helsinki and the International Conference of Harmonization of Good Clinical Practice guidelines. We included SD-OCT data of 44,903 SD-OCT volume scans from 2771 patients with nAMD enrolled in the prospective double-masked, multicentre, active-controlled, randomized clinical trials HAWK (clinicaltrials.gov identifier NCT02307682 and HARRIER (clinicaltrials.gov identifier NCT02434328). The sponsor of each respective trial provided the data, but the analyses, content and conclusions presented here are solely the responsibility of the authors.

Treatment and imaging protocol for the studies have been presented previously [14]. In these studies, the OCT scans were acquired with devices used in clinical routine: Cirrus - Zeiss Meditec, Spectralis – Heidelberg Engineering, Topcon-1000 and Topcon-2000.

### Automated deep learning-based analysis

These were then analysed using a previously published [15] deep learning algorithm trained on Cirrus and Heidelberg systems to detect and localize retinal fluid by compartment and to quantify intraretinal fluid (IRF) and subretinal fluid (SRF) volumes. This was computed for the central 1, 3, and 6 millimetres of the macula. The measured volumes of the algorithm were then compared to the expert grading status of fluid (presence or absence) from the Vienna Reading Center in HARRIER and Duke Reading Center in HAWK.

To measure retinal thickness, we employed the Iowa Reference Algorithms (Retinal Image Analysis Lab, Iowa Institute for Biomedical Imaging, Iowa City, IA) to automatically delineate inner limiting membrane (ILM) and outer RPE boundaries of the retina. Bruch’s membrane (BM) was automatically delineated using an in-house designed convolutional neural network trained to segment a smooth BM boundary on retinal OCT scans [16]. We defined CSFT as the mean thickness of the retina in the central 1 mm between the ILM and the outer RPE boundary.

### Human expert grading

To evaluate central retinal thickness in its entirety, we compared both CPT and CSFT measurements collected by the Vienna Reading Center (both ILM to RPE inner surface and ILM to Bruchs’ membrane (BM)) with measurements made by the algorithm. This analysis was done in a subsection of patients using 50 baseline and 50 follow-up visits of different, randomly chosen eyes with OCT-device distribution being proportional to their prevalence in the respective studies (Spectralis 67%, Cirrus 29%, and Topcon 4%). Due to the low number of Topcon scans, these were excluded in this analysis.

### Statistics

If not indicated otherwise, all measurements were analysed separately at baseline and follow-up visits. For the fluid volumes and CSFT values, the median, and interquartile range are reported. Spearman’s correlation coefficient (ρ) was used to examine the associations between fluid volumes and CSFT as well as CSFT with expert grading. We utilized Cohen’s terminology to describe the magnitude of correlation (ρ): 0.20 ≤ ρ < 0.5 small; 0.50 ≤ ρ < 0.7 moderate; ρ ≥ 0.70 large [17].

Additionally, linear regressions for CSFT and SRF height explained by fluid volume were performed to determinate the residual standard deviations at baseline. For the follow-up visit measurements, the linear mixed models for CSFT explained by fluid volume with random effects were used to report the marginal residual standard deviations.

Statistical analyses were performed in R version 4.1.0.

## Results

**Fig. 1:**
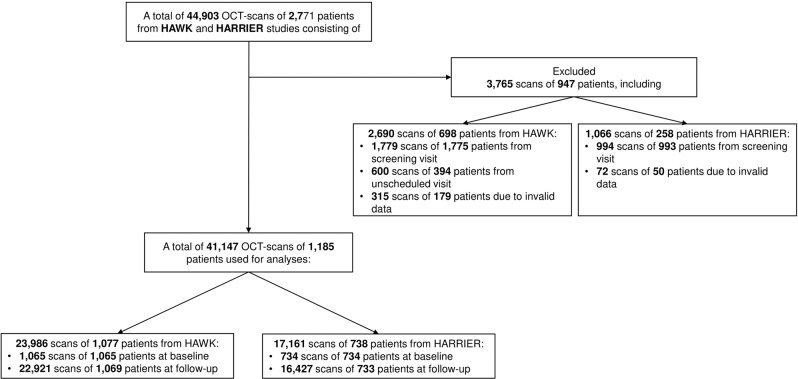
Flow diagram of scans processing. 44,903 OCT scans were available from HAWK and HARRIER studies. 3.765 scans had to be excluded leaving 41,147 scans of 1,185 patients for analysis.

Figure 1 demonstrates a total of 44,903 SD-OCT volume scans from 2771 patients that were initially available. 3765 scans were excluded due to being from screening or unscheduled visits or due to missing data.

### Detection of fluid presence/absence by human versus automated assessment

**Fig. 2:**
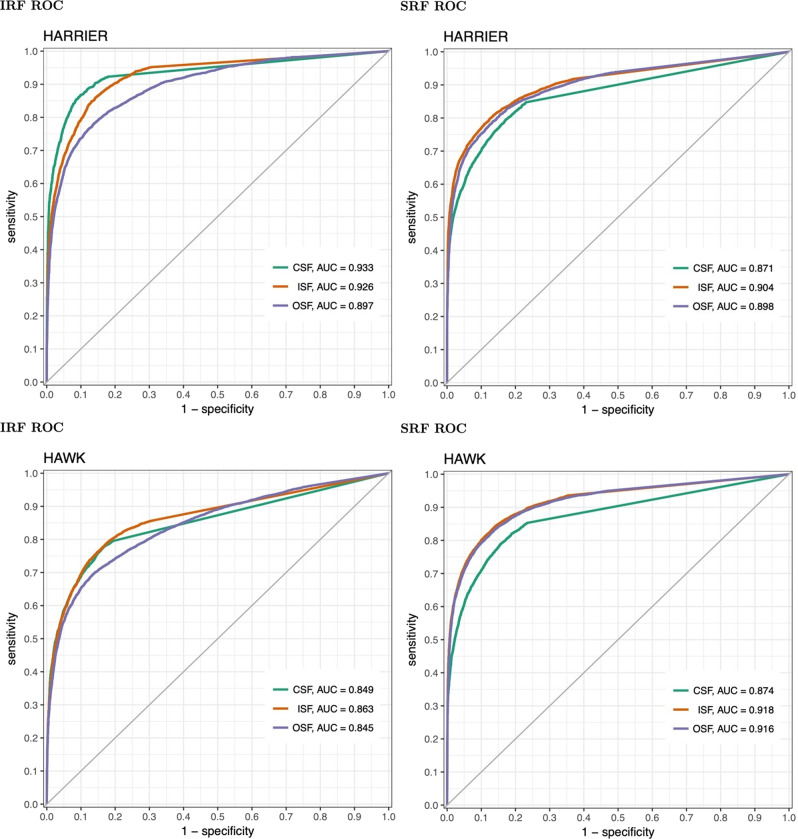
Detection level AUC values of HARRIER and HAWK. AUC values of the detection of fluid between expert graders and algorithmic reading in HAWK and HARRIER.

We analysed 17,161 OCT scans from monthly visits over two years in HARRIER and 23,986 OCT monthly scans in HAWK. In the central millimetre, the concordance for the detection of fluid between expert grading and algorithmic reading reached a value of 0.93 for IRF and 0.87 for SRF in HARRIER, see Fig. 2. Concordance for the detection of fluid by compartment between expert readers and algorithm reached an AUC of 0.85 for IRF and 0.87 for SRF in the central millimetre in HAWK. In the central 3 mm, AUC values were 0.92 for IRF and 0.90 for SRF in HARRIER and 0.86 for IRF and 0.91 for SRF in HAWK. In the central 6 mm, the concordance for IRF and SRF both reached an AUC of 0.90 in HARRIER and 0.85 for IRF and 0.91 for HAWK. The highest concordance was reached on scans from the Spectralis device with an AUC of 0.93 followed by Cirrus and Topcon images both with an AUC of 0.90 for IRF when pooling all areas. For the detection of SRF, Spectralis also had the best AUC of 0.91 followed by Topcon (AUC 0.87) and Cirrus (AUC 0.86) in HARRIER. Across all three areas, the concordance between algorithm and reading centre was similar for IRF detection across both Spectralis, AUC of 0.85, and Cirrus, AUC of 0.88 in HAWK. For the detection of SRF, Spectralis had a higher AUC of 0.91 compared to Cirrus with 0.89. AUC values per fluid compartment and instrument are reported in Table 1. Segmentation examples can be found in the Supplementary Materials.

**Table. 1:** Comparison of AUC values per fluid compartment and instrument.

Fluid	Instrument	HAWK	HARRIER
IRF	Cirrus	0.882	0.904
	Spectralis	0.848	0.931
	Topcon	NA	0.900
SRF	Cirrus	0.891	0.864
	Spectralis	0.909	0.911
	Topcon	NA	0.869
PED	Cirrus	0.704	0.696
	Spectralis	0.684	0.671
	Topcon	NA	0.642

### Quantification of fluid volume resolution

**Fig. 3:**
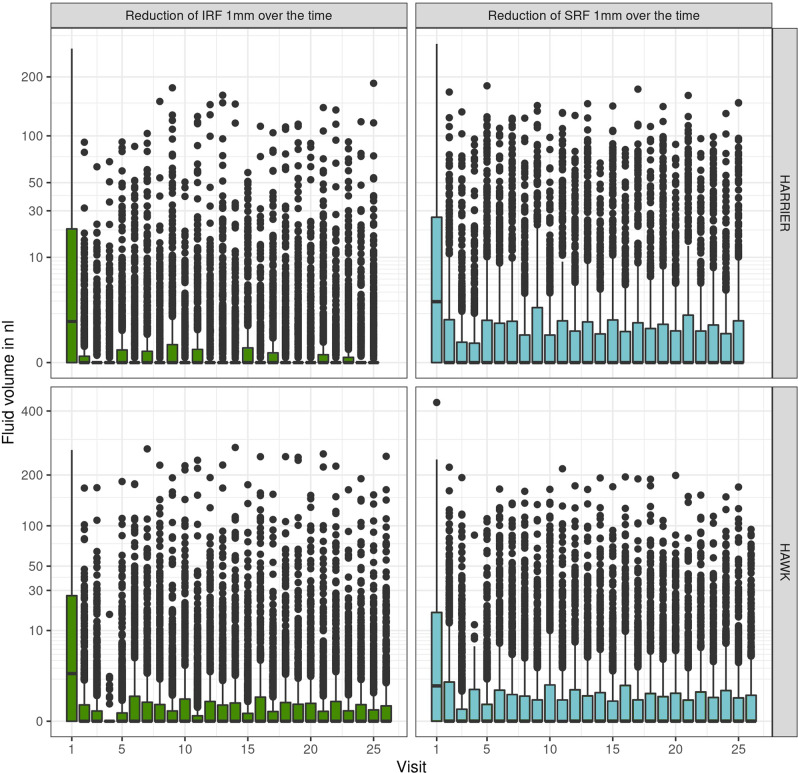
Median fluid volumes (IRF and SRF) with interquartile range over time.

The quantified monitoring of fluid volumes over time allows an objective overview about fluid resolution patterns and disease activity (Fig. 3). IRF volumes were primarily low, reacted immediately to treatment and remained low with little fluctuations. Also, SRF showed a pronounced resolution after the first injection, but demonstrated rather persistent presence and quantity over the two years of monitoring Overall, the therapeutic response measured on the base of compartments and volumes was consistent over all time points and reflected disease activity as well as the behaviour of the clinical investigators in their decision-making.

### Comparison of fluid volumes and CSFT

We analysed 734 baseline visits in HARRIER and macular IRF volume showed a moderate correlation with CSFT at 1 mm: ρ = 0.510, 3 mm: ρ = 0.507 and 6 mm: ρ = 0.508. The association of SRF with CSFT at baseline was very weak for 1 mm: ρ = 0.091, 3 mm: ρ = 0.089 and 6 mm: ρ = 0.146.

**Fig. 4:**
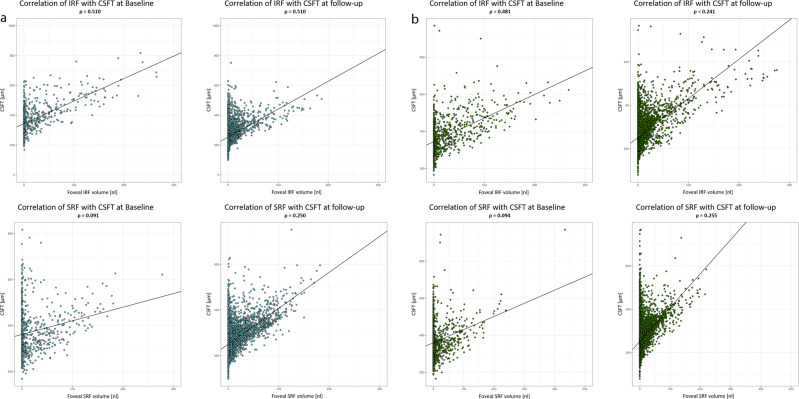
**a** Correlation of fluid volumes (IRF and SRF) at baseline and under therapy with CSFT in HARRIER´. **b** Correlation of Fluid volumes at baseline and under therapy with CSFT in HAWK.

16,427 follow-up visits were analysed to highlight the value of CSFT measurements as treatment guidance during monitoring. The correlation of IRF and CSFT was weak under therapy; 1 mm: ρ = 0.250 | 3 mm: ρ = 0.289 | 6 mm: ρ = 0.278. Association of SRF with CSFT improved only marginally; 1 mm: ρ = 0.305 | 3 mm: ρ = 0.363 | 6 mm: ρ = 0.361 (Fig. 4a).

The residual standard error at baseline (IRF: 75.90 µm; SRF: 95.26 µm) and the marginal residual standard deviations at follow-up (IRF: 46.35 µm; SRF: 44.19 µm) of fluid volume were high compared to the range of CSFT values, reflecting the inaccuracy of CSFT to determine fluid volumes and their fluctuation.

We analysed 1065 baseline visits from the HAWK trial. Consistently, IRF volumes showed only a moderate association with CSFT at baseline for 1 mm: ρ = 0.481, 3 mm: ρ = 0.459 and 6 mm: ρ = 0.447. The correlation of SRF and CSFT was weak at baseline for 1 mm: ρ = 0.094, 3 mm: ρ = 0.119 and 6 mm: ρ = 0.199. When analysing the 22,921 follow-up visits in HAWK, IRF had an even weaker correlation with CSFT under therapy than at baseline for 1 mm: ρ = 0.241, 3 mm: ρ = 0.271 and 6 mm: ρ = 0.251. The low association of SRF with CSFT did not increase much under therapy for 1 mm: ρ = 0.255, 3 mm: ρ = 0.305 and 6 mm: ρ = 0.302 (Fig. 4b).

The residual standard error, i.e. the variability of individual fluid volumes at baseline (IRF: 78.47 µm; SRF: 89.19 µm) and the marginal residual standard errors at follow-up (IRF: 45.43 µm; SRF: 46.19 µm) of fluid volume were high compared to the range of CSFT values.

### Correlation of manual and automated CSFT

The correlations of CSFT measured using automated algorithms were high with all thickness values measured by the Vienna Reading Center. (i.e. CSFT and CPT measured both from ILM to BM and ILM to RPE). However, the correlation was more robust for CSFT values from ILM to RPE (CPT ρ = 0.916; CSFT ρ = 0.942) compared to the enlarged CSFT definition where RPE detachments are included (CPT ρ = 0.786; CSFT ρ = 0.819).

## Discussion

When treating patients with anti-VEGF drugs, the goal is to optimize therapeutic benefit while minimizing treatment burden. With the change from early fixed monthly treatment regimens to flexible, i.e. monitoring-driven pro re nata (PRN) and treat-and-extend strategies, rewards such as patient compliance, reduced cost and fewer injection related complications are a huge benefit [7, 18, 19]. Accordingly, these personalized regimens rely heavily on an accurate identification of retinal fluid as the decisive guiding parameter for treatment intervals. CSFT was introduced in the era of time-domain OCT imaging when only 6 radial scans were available, but continued through the advent of spectral domain OCT using raster scanning which creates image volumes. The semi-quantitative parameter is still used in large scale randomized clinical trials as primary or secondary outcome measure to reflect exudative activity, as measuring retinal fluid has so far not been feasible [20, 21]. But even when just detecting the mere presence of macular fluid Toth et al. showed in CATT that there is a substantial discrepancy between ophthalmologists and trained RC personnel in 27.9% of cases. Over 90% of discrepancies were due to the detection of fluid in OCT by RC in patients who were not treated by ophthalmologists [22]. In the literature it is surmised that the disappointing real-world outcomes of anti-VEGF therapy in current clinical practice are largely explainable by undertreatment in this setting further highlighting the need for accurate fluid detection [23]. We know that eyes with residual fluid in the retina have worse visual acuity (9 letters in CATT) [24] and that progressive intraretinal cyst formation makes BCVA loss more pronounced and irreversible [25]. Recent studies with data from large scale phase III trials have shown an association of IRF per 100 nl of volume in the central mm with a reduction of BCVA between −2.8 and −4.08 letters [26, 27]. In addition, a real-world analysis from routine nAMD management, the VIBES study, has shown that IRF is the most important indicator for visual acuity correlations over as long as 5 years considering IRF, SRF and CSFT [28]. It has also been shown that SRF may not only be associated with better baseline and outcome BCVA [29], but also with a lower risk for geographic atrophy which further underscores the need for reliable fluid localization and quantification [30]. With its location being largely beyond the central 1 mm and therefore being spatially separated from central IRF, the absolute amount of SRF is largely underestimated [31].

The task of our study was to comprehensively assess fluid management by comparing the accuracy and outcomes of human and automated procedures including the related parameters, semi quantitative 1D CSFT versus pixel-based 3D volumes, in a phase of transition of available procedures. Our results show that deep learning-based image evaluation is able to bridge the current gap using automated algorithms convincingly with AUC values for the detection of IRF reaching as high as 0.933 in the central millimetre, where IRF is most relevant and 0.916 for SRF in the central 6 mm, where SRF is predominantly located. This was true for all of the OCT devices used in both studies even though the algorithm was not validated on Topcon devices. In terms of detecting the presence of IRF and SRF in OCT scans, the computerized analysis achieved accuracies in the range of inter-observer agreement between certified reading experts reported in the literature [32]. Such human expert readers are trained and certified for specific tasks in regulatory approval studies and are spending hours on image segmentation, in contrast to the fast turn-over clinicians in routine practice have to deliver. Overload and burn-out of physicians in this heavy duty of nAMD care is widely discussed and has even been included in programs of large congresses such as the AAO. Furthermore, computerized annotation has the advantage of not only segmenting and measuring retinal compartments at all time points, but alerting clinicians to details they might otherwise miss in the large 3D fly-throughs.

The diagnostic tasks in nAMD care include a comprehensive evaluation of disease activity at first presentation, i.e. baseline, and a time-sensitive and precise long-term monitoring of recurrence and therapeutic response thereafter. When assessing fluid volumes and CSFT at baseline, we observed only moderate to small correlations despite substantial amounts of retinal fluid present in the untreated macula. It has already been shown that the large quantities of SRF are not limited to the foveal region, but are mostly located around the central IRF. Distinct SRF pooling is therefore primarily unlikely to be represented by the one-dimensional marker that is CSFT [31]. Apart from intra- and subretinal fluid, there are many other pathomorphologic components such as neovascular membranes, drusen, subretinal hyperreflective material (SHRM) or fibrovascular PED that may affect CSFT during the course of the disease. During monitoring, the correlation of IRF and CSFT was further reduced under anti-VEGF therapy, while the association of SRF and CSFT remained consistently low. Under treatment, patients lost 89.9–93.2% of mean IRF and 81.7–84.2% of mean SRF compared to baseline with only tiny amounts of fluid remaining in the retina. Despite therapy with anti-VEGF and even in the absence of exudation, patients are at risk of developing both fibrosis and atrophy which affect CSFT in opposite ways. Such structural changes may play a bigger role in respect to thinning and thickening of the retina. Figure 4a and b further support this observation, as patients with nil to low fluid values show a broad range of central retinal thickness values. Therefore, CSFT seems to be insufficient in its function as surrogate for exudative activity in patients with nAMD as suggested by prior publications [28]. Once the retina thins under therapy, it is plausible that fluctuations due to increasing or decreasing SRF volume are more important which might explain the increasing correlation of CSFT with SRF. Noteworthy, based on CSFT values, the FLUID study comparing a tight versus a relaxed SRF treatment regimen concluded that SRF may be left untreated if below a certain CSFT level. However, a deep learning-based analysis of fluid volumes revealed that the two study arms did not present any difference in SRF volumes from the beginning and that increasing SRF volumes did indeed lead to functional loss [33]. Very high residual standard deviations at baseline (IRF: 75.90 and 78.47 µm, SRF: 95.26 and 89.19) and the marginal residual standard deviations at (IRF: 46.35 and 45.43 µm; SRF: 44.19 and 46.19) follow-up were found compared to the range of CSFT values. Values of such magnitude impressively highlight that retinal thickness and fluid amounts provide different information, and treatment decisions regarding fluid should not be based on thickness only. This is particularly relevant as in many recent trials for regulatory approval of novel substances introducing longer-acting compounds the retreatment intervals are based on CSFT [34, 35]. Moreover, the changes in CSFT triggering retreatment vary widely from 25 µm to 75 µm as compared to previous best or averaged CSFT values which has a strong impact on the determination of the treatment-free intervals. In the study protocol of HAWK and HARRIER which we used in our analyses, extension of retreatment intervals was also the major focus. The retreatment administration according to the investigators’ performance resulted in variable amounts of recurrence and residual fluid, see Fig. 3 which became apparent when volumes were measured by automated AI-based analyses.

In terms of fluid/function correlation, it has already been shown that the presence of IRF and SRF has different ramifications for the retina. While SRF seems to be correlated with a better visual outcome at baseline visits and seems to have a better tolerance towards infrequent treatment regimens [29], IRF is strongly correlated with lower VA at baseline and under anti-VEGF therapy at all timepoints [28, 29, 36]. Furthermore, it has been shown that the vertical extension of IRF is only relevant up to a threshold of 20 µm with regards to visual function further highlighting the shortcoming of CSFT as a biomarker for patients with nAMD as only the verticality is represented by thickness values [36]. Hence, fluid volumes, dynamics and timing matter greatly for maintaining function in anti-VEGF therapy of nAMD.

Limitations of the study are its retrospective nature, yet, no prospective trials using automated fluid volume quantification have been conducted so far as the deep learning tools are just becoming available. It will be most revealing to understand the impact of a volume-driven in contrast to a conventional CSFT-triggered regimen on retreatment frequency and particularly visual outcome. The population size and image quantity with more than 40.000 scan volumes in our study is large enough to provide relevant results including those retrieved from statistical deviations, although these data should be used for further evaluation instead of final conclusions at this stage. A further limitation is that the algorithm was developed and validated using Spectralis, Cirrus and Biotigen systems but not on Topcon devices. However, Topcon scans contributed less than 5% of data and reported performance AUC values are similar between devices, meaning that the model seems to generalize well.

Our study demonstrates that the identification of fluid volumes and central retinal thickness values from automated deep learning-based segmentation of retinal OCT scans reached high concordance with expert grading. AI tools can perform at the level of certified reading professionals and even beyond human inter-reader variability, safe time to achieve precision results and provide objectivity in monitoring patients not only in clinical trials, but particularly real-world practice. Furthermore, automated quantification of nAMD features across the entire macula, beyond retinal thickness measurements, illustrates the potential of such approaches to realistically analyse disease activity and therapeutic response in order to optimally support robust treatment decision in nAMD patients in clinical routine. Automated and accurate quantification of the fluid response should improve the therapeutic management of nAMD by avoiding subjective variability between clinicians/investigators, establishing a reliable structure/function correlation and lead to the development of advanced management protocols. Prospective clinical studies will have to be performed to provide future evidence of such a point-of-care concept. In the future, these tools will be important guides for treatment decisions on intervals, substances and monitoring needs and should supplement or replace retinal thickness measurements allowing to deliver the diagnostic benefit of high-resolution three-dimensional imaging as a therapeutic benefit to our patients.

## Summary

### What was known before

- Many recent trials for regulatory approval of novel substances introducing longer-acting compounds base retreatment intervals on CSFT.

### What this study adds

- Retinal thickness and fluid amounts provide different information, and treatment decisions regarding fluid should not be based on thickness only. Retinal thickness does not provide reliable information on fluid volume and vice versa. Deep learning-based image evaluation is able to bridge this gap using automated algorithms convincingly.

### Supplementary information

Supplemental Materials

## Data Availability

Original data for this research were provided by Novartis Pharma AG. Data that support the findings of this study are available upon reasonable request.
